# Insulin-stimulated brain glucose uptake correlates with brain metabolites in severe obesity: A combined neuroimaging study

**DOI:** 10.1177/0271678X231207114

**Published:** 2023-10-12

**Authors:** Eleni Rebelos, Aino Latva-Rasku, Kalle Koskensalo, Laura Pekkarinen, Ekaterina Saukko, Jukka Ihalainen, Miikka-Juhani Honka, Jouni Tuisku, Marco Bucci, Sanna Laurila, Johan Rajander, Paulina Salminen, Lauri Nummenmaa, Jacobus FA Jansen, Ele Ferrannini, Pirjo Nuutila

**Affiliations:** 1 Turku PET Centre, 8058 University of Turku, Turku, Finland; 2 Institute of Clinical Physiology, National Research Council (CNR), Pisa, Italy; 3 Department of Clinical and Experimental Medicine, University of Pisa, Pisa, Italy; 4 Department of Endocrinology, Turku University Hospital, Turku, Finland; 5 Department of Medical Physics, Turku University Hospital, Turku, Finland; 6 Department of Radiology, Turku University Hospital, Turku, Finland; 7 Turku PET Centre, Accelerator Laboratory, Åbo Akademi University, Turku, Finland; 8 Theme Inflammation and Aging, Karolinska University Hospital, Stockholm, Sweden; 9 Division of Clinical Geriatrics, Center for Alzheimer Research, Department of Neurobiology, Care Sciences and Society, Karolinska Institutet, Stockholm, Sweden; 10 Department of Digestive Surgery and Urology, Turku University Hospital, Turku, Finland; 11 Department of Surgery, University of Turku, Turku, Finland; 12 Department of Psychology University of Turku, Turku, Finland; 13 Department of Radiology, Maastricht University Medical Center, Maastricht, The Netherlands

**Keywords:** Positron emission tomography, magnetic resonance spectroscopy, insulin resistance, obesity, N-acetyl-aspartate, myo-inositol

## Abstract

The human brain undergoes metabolic adaptations in obesity, but the underlying mechanisms have remained largely unknown. We compared concentrations of often reported brain metabolites measured with magnetic resonance spectroscopy (^1^H-MRS, 3 T MRI) in the occipital lobe in subjects with obesity and lean controls under different metabolic conditions (fasting, insulin clamp, following weight loss). Brain glucose uptake (BGU) quantified with ^18^F-fluorodeoxyglucose positron emission tomography (^18^F-FDG-PET)) was also performed in a subset of subjects during clamp. In *dataset A,* 48 participants were studied during fasting with brain ^1^H-MRS, while in *dataset B* 21 participants underwent paired brain ^1^H-MRS acquisitions under fasting and clamp conditions. In *dataset C* 16 subjects underwent brain ^18^F-FDG-PET and ^1^H-MRS during clamp. In the fasting state, total N-acetylaspartate was lower in subjects with obesity, while brain myo-inositol increased in response to hyperinsulinemia similarly in both lean participants and subjects with obesity. During clamp, BGU correlated positively with brain glutamine/glutamate, total choline, and total creatine levels. Following weight loss, brain creatine levels were increased, whereas increases in other metabolites remained not significant. To conclude, insulin signaling and glucose metabolism are significantly coupled with several of the changes in brain metabolites that occur in obesity.

## Introduction

Both obesity and type 2 diabetes (T2D) have been linked with an increased risk for neurodegeneration and Alzheimer’s disease (AD).^1–3^ While the prevalences of obesity and T2D are rapidly increasing, aging of the population is the greatest risk factor for neurodegeneration.^4^ Thus, better understanding of brain physiology and the brain-specific pathophysiology of metabolic disorders is taking on a new urgency. However, the human brain remains the least accessible of organs, and *in vivo* attempts to study its physiology rely predominantly on neuroimaging.^5–13^

In a compelling contrast to peripheral tissues, we have previously shown consistently that when studied with positron emission tomography (PET) using the glucose analogue tracer ^18^F-fluorodeoxyglucose (^18^F-FDG),^14^ brain glucose uptake (GU) is increased in subjects with severe obesity or insulin resistance during hyperinsulinemic, euglycemic clamp. Surprisingly, under fasting conditions the brain GU rates do not differ between these subjects and healthy, lean controls, who in contrast show no acceleration in GU during hyperinsulinemic euglycemia. This finding has been later confirmed by others in both humans and animals.^7,15^ Alarmingly, this metabolic disruption seems to initiate early, already in subjects at risk or at the very early stages of metabolic syndrome.^16^ In the largest cohort by far of 194 subjects studied with ^18^F-FDG-PET during hyperinsulinemic, euglycemic clamp, we showed that peripheral insulin sensitivity is highly correlated with body mass index (BMI), which is also the best single predictor of brain GU.^9^ However, the molecular mechanisms that underlie this finding remain elusive.

Proton magnetic resonance spectroscopy (^1^H-MRS) is a neuroimaging method that quantifies metabolite concentrations in the tissue of interest, and it has been used in several previous studies to characterize changes occurring in the brain in obesity.^17,18^ However, in the brain measuring glucose concentration or metabolic fluxes is less candid due to low natural concentration of glucose in the brain and the overlap with more prominent spectral peaks, whereas ^18^F-FDG-PET can provide an explicit, direct quantitative assessment of glucose uptake in the tissue. Considering the poor accessibility of the human brain, both neuroimaging modalities are extremely useful as they provide complementary information.

Thus, to understand better the increased insulin-stimulated brain GU in severe obesity, we performed a series of brain ^1^H-MRS studies assessing concentrations of different metabolites often reported altered in obesity and insulin resistance: total N-acetyl-aspartate (NAA), myo-inositol, glutamine/glutamate (Glx), total creatine and total choline. We conducted the ^1^H-MRS studies under both fasting and hyperinsulinemic, euglycemic clamp conditions to determine the effects of obesity, as previously done by us and others to assess BGU,^7,19^ and tested whether during insulinized conditions the intracerebral concentrations of these metabolites associate with increased brain GU. The effect of weight loss on brain metabolites was also assessed with a ^1^H-MRS scan 1-year postoperatively in a subset of subjects with severe obesity.

Considering that some studies have suggested that astrocytes take up most of cerebral ^18^F-FDG,^20^ we initially hypothesized that the increase in BGU would be associated with astrocytosis,^21,22^ and that in ^1^H-MRS this change would be measurable as an increase in the glial marker myo-inositol.^23^ Additionally, we expected the increase in BGU to coincide with a decrease in NAA, a marker of neuronal viability, and insulin stimulation to affect the levels of glutamate/glutamine.^24,25^

## Materials and methods

### Study design

**Figure 1.**
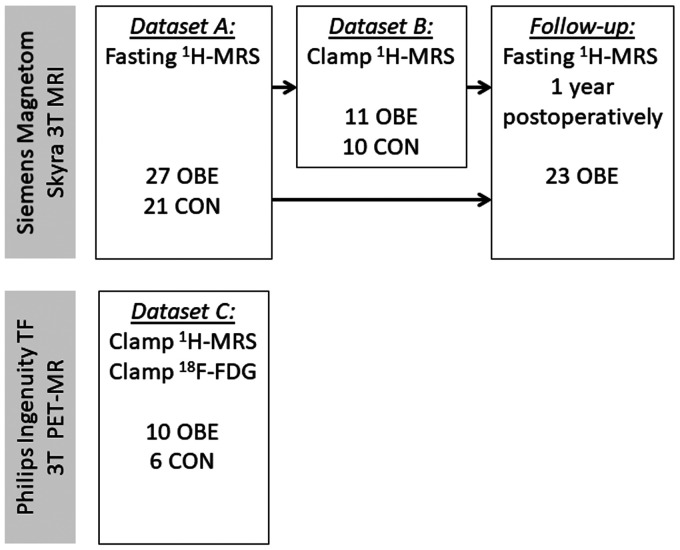
Study flowchart.

A total of 37 subjects with severe obesity (OBE) undergoing evaluation for bariatric surgery in the Department of Digestive Surgery and Urology, Turku University Hospital (Turku, Finland) were recruited for the studies, along with 27 age- and gender-matched healthy lean controls (CON) recruited by ads in local newspapers and the hospital website. In the first study (dataset A), effects of severe obesity on brain metabolites concentrations were evaluated by fasting brain ^1^H-MRS. Part of the subjects in dataset A also underwent the ^1^H-MRS studies during hyperinsulinemic, euglycemic clamp to investigate the effects of insulin (dataset B). Due to MRI equipment update, the association between brain ^1^H-MRS and ^18^F-FDG-PET-derived GU was only evaluated in a separate group of subjects (dataset C). The flowchart of the study is presented in Figure 1.

Studies were conducted before the OBE subjects started a 4‐week very‐low‐energy dietary regime prior to bariatric surgery. Strenuous physical activity was prohibited from the preceding evening and all antidiabetic drugs were withheld 72 h before the studies.

### Participants

For dataset A, comprising of fasting ^1^H-MRS studies, 27 OBE and 21 CON subjects were recruited. Of these subjects, 11 OBE and 10 age- and sex-matched CON also participated in the insulin-stimulated ^1^H-MRS-studies (dataset B). A separate group of 16 subjects (10 OBE and 6 CON) underwent ^1^H-MRS and ^18^F-FDG-PET/CT studies during hyperinsulinemia (dataset C). 23 out of 27 patients of dataset A were re-studied ∼1 year following bariatric surgery.

All study participants were free of previous neurological disease (*e.g*., cognitive impairment, large vessel stroke, seizure disorder, Parkinson’s disease, clinically significant traumatic brain injury, multiple sclerosis, or previous brain infection/meningitis), and none of them had any major psychiatric illnesses (*e.g*., schizophrenia, bipolar disorder) or reported substance abuse. Subjects were also eligible to undergo magnetic resonance imaging. After giving written informed consent, all subjects underwent a screening visit where basic anthropometric and clinical measurements were obtained (body weight, height, waist and hip circumferences, blood pressure). Body fat mass percentage was measured with an air displacement plethysmograph (the Bod Pod system, software version 5.4.0, COSMED, Inc., Concord, CA, USA) after at least four hours of fasting. Blood tests comprised a standard (75 g) oral glucose tolerance test (OGTT) with frequent blood sampling at 0, 30, 60, 90 and 120 minutes for determination of glucose, insulin, and C‐peptide. For the CON group, previously diagnosed T2D or OGTT results indicating T2D were considered an exclusion criteria, while subjects with impaired glucose tolerance with or without impaired fasting glucose based on the ADA criteria^26^ could be enrolled. In the OBE group subjects with T2D had been previously prescribed metformin and/or dapagliflozin, but no other treatments affecting glucose metabolism. To avoid possible confounding effects of gender in a small sample, only female participants were studied in dataset C, while both male and female subjects were enrolled in datasets A and B.

### Brain ^1^H-MRS studies

Fasting studies (dataset A) were performed after an overnight fast. On visits studying the effects of insulin (dataset B), ^1^H-MRS studies were first performed during fasting, and then repeated 120 ± 10 min into hyperinsulinemic, euglycemic clamp, which was performed as previously described.^27^ In brief, a primed-continuous infusion of insulin (Actrapid; Novo Nordisk, Copenhagen, Denmark) was given at a rate of 40 mU.m^−2^.min^−1^. During the clamp, a 20% glucose solution was infused at a rate designed to maintain euglycemia (∼5 mmol/L). Plasma glucose levels were measured every 5–10 min throughout the clamp, plasma insulin and serum free-fatty acid concentrations were taken at baseline and every 30 and 60 min, respectively. In the dataset evaluating potential correlations between brain metabolites and brain GU (dataset C), subjects first underwent an ^18^F-FDG-PET/CT scan (brain scanned ∼ 95 min into the clamp), and at ∼150 min into the clamp they were placed in the MRI scanner, and the brain ^1^H-MRS measurement was acquired thereafter.

**Figure 2.**
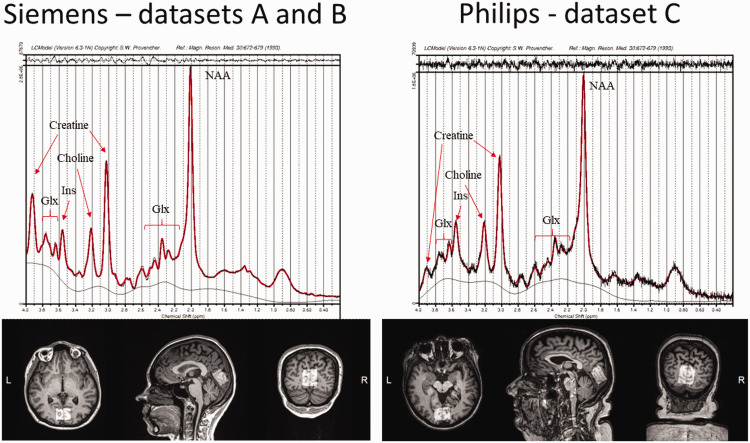
Voxel placement for 1H-MRS in the occipital lobe, and representative examples of fitted spectrums in the two scanners. Glx: glutamate/glutamine; Ins: myo-inositol; NAA: total N-acetyl-aspartate.

Subjects were studied with either a Siemens Magnetom Skyra fit 3 T MRI scanner (Siemens Medical Solutions, Erlangen, Germany) with 20-channel Head/Neck Matrix coil (datasets A and B), or with a Philips Ingenuity TF 3 T PET-MR scanner (Philips Healthcare, Cleveland, OH, USA) with a 32-channel SENSE Head coil (dataset C). Subjects were positioned supine inside the MRI and the head was immobilized with foam inserts on top of a radiofrequency probe. After a brain morphology scan, ^1^H-MRS spectrum was measured from a 20*x*30*x*30 mm voxel in the center of the occipital lobe using PRESS sequence with 176 water-suppressed signal averages and 4 signal averages without water suppression. The occipital lobe was selected for reliable spectral acquisition and to enable result comparisons to previous reports.^20,21^ The voxel was placed in the center of the occipital lobe covering parts of both hemispheres, making sure that the margins of the voxel would not be close to the skull or the cerebellum (Figure 2). All voxel placements were performed by a single experimenter (K.K.). In the paired brain ^1^H-MRS screen shots of the first voxel placement were used as a reference for the voxel placement in the second scanning. The measurement lasted for 10 minutes with the sequence parameters repetition time = 3000 ms, echo time = 35 ms, bandwidth = 2000 Hz. Water suppression was performed using the CHESS method. The acquisition parameters of the ^1^H-MRS experiments are given in Table 1.

**Table 1.** Acquisition parameters of the ^1^H-MRS.

	Siemens	Philips
Volume selection method	SVS	
Voxel size RL × AP × FH	30 mm × 20 mm × 30 mm	
Sequence type	PRESS	
Repetition time	3000 ms	
Echo time	35 ms	
Bandwidth	2000 Hz	
Averages	176	
Non-water suppressed averages	4	16
Time of acquisition	9 min 12 s	9 min 42 s
Water suppression method	CHESS	Excitation
Water suppression bandwidth	35 Hz	200 Hz
Number of data points	2048	4096
Dataset studied	A and B	C

SVS: Single Voxel Spectroscopy; PRESS: Point Resolved Spectroscopy Sequence; RL: right-left; AP: anterior-posterior; FH: feet-head.

### Quantification of ^1^H-MRS data

Eddy current corrected ^1^H-MRS data were analyzed using LCModel (Version 6.3-1 N),^30^ and water scaling was performed to obtain metabolite concentrations in mM units. Therefore, in addition to the water suppressed spectrum, LCModel was given also a non-water-suppressed spectrum to scale the metabolite signals in relation to the water signal. The analysis window was set from 4.0 to 0.2 ppm. A simulated basis set including 21 model metabolite signals and 13 stimulated lipid and macromolecule signals was used.^31^ After metabolite concentration values were produced with the default parameters of the LCModel software (assuming water concentration of 35880.0 mM), brain tissue fractions (grey matter, white matter, cerebrospinal fluid) were derived for more accurate estimation of the concentrations of water, and this was used as an internal reference. The tissue fractionation was performed with the use of Gannet software tool (Version 3.3.1)^32^ as follows: first the acquired MRS voxel data was co-registered to the MRI image using the SMP12 software (https://www.fil.ion.ucl.ac.uk/spm/software/spm12/) and then segmented to fractions of three compartments: grey matter, white matter and cerebrospinal fluid.^33^ Their respective water concentration was assumed to be 43300 mmol/L, 35880 mmol/L and 55556 mmol/L.^34^ Thus, a more accurate estimate of the water concentration within the voxel was achieved by multiplying the concentration of water in each compartment by the fraction of each compartment and the metabolite concentrations were corrected to be in relation to this value. The T1/T2 relaxation corrections in different compartments to different metabolites was not performed due to uncertainties in relaxation times. However, as acquisition parameters were kept constant during the study, relaxation times were not expected to change.

### Brain ^18^F-FDG-PET/CT protocol

To acquire insulin-stimulated brain GU (dataset C), a hyperinsulinemic, euglycemic clamp was started after an overnight fast and continued throughout the scan as described above. Subjects were placed into a GE Advance PET/CT camera (General Electric Medical Systems, Milwaukee, Wisconsin), and 70 ± 10 min into the clamp, ^18^F-FDG (187 ± 9 MBq) was injected intravenously followed by a 10-minute static acquisition of brain radioactivity at 95 ± 6 min into the clamp. All data were corrected for dead time, decay, and measured photon attenuation, and reconstructed using a Hann filter with a cut‐off frequency of 0.5 and a median root prior reconstruction method. Plasma radioactivity used for input function was measured from arterialized venous blood samples collected during the scan and analyzed using an automatic γ counter (Wizard 1480; Wallac, Turku, Finland).

### Quantification of brain glucose uptake

Fractional uptake rate (FUR),^35^ the quotient of tissue activity divided by the integral of plasma activity from injection until the middle of the selected frame, was calculated for each voxel separately. Next, voxelwise GU (µmol/kg/min) was calculated using the formula GU = (FUR × Cp)/(LC^ × ^tissue density) × 100, where Cp is the average plasma glucose concentration from the injection until the end of the brain scan, and LC is the lumped constant for the brain (set at 0.65).^36^ Brain density was set at 1.04.^8^ Summed PET images were spatially normalized to the ^18^F-FDG template in Montreal Neurological Institute space (MNI International Consortium for Brain Mapping) using statistical parametric mapping (SPM [SPM12; www.fil.ion.ucl.ac.uk/spm/]) running on Matlab for windows (version 9.1.0; Math Works, Natick, Massachusetts). Normalization variables were subsequently applied to corresponding parametric glucose metabolism images. Parametric images were smoothed at 10 mm full‐width at half‐maximum. Occipital lobe GU was obtained using two toolboxes for Matlab: WFU_PickAtlas (https://www.nitrc.org/projects/wfu_pickatlas) to generate the VOI mask of interest (http://www.talairach.org/daemon.html) and Marsbar (http://marsbar.sourceforge.net) to extract the GU values from the parametric images after applying the VOI mask.

### Insulin-stimulated glucose disposal (M value)

The *M* value was calculated during the second hour of the clamp as a mean of three 20 min intervals and expressed per kg of fat-free mass (μmol. kg_FFM_^−1^. min^−1^), as this normalization has been shown to minimize differences due to sex, age, and body weight.^37^

### Analytical methods

Plasma glucose was measured in the laboratory of Turku PET Centre in duplicate using the glucose oxidase technique (Analox GM7 or GM9, Analox Instruments Ltd., London, UK). Glycosylated haemoglobin (HbA_1c_) and plasma insulin were measured with automated electrochemiluminescence immunoassay (Cobas 8000; Roche Diagnostics, Mannheim, Germany).

### Statistical analysis

Data are presented as mean ± SD, or median [IQR] for non-normally distributed variables. Between groups comparisons were performed by *t*-test or Wilcoxon rank-sum test as appropriate. The Shapiro-Wilk test was used to assess normality. Comparisons between categorical variables was performed by the χ^2^ test. Correlations between brain metabolites and GU were analysed using Pearson’s *r*, and confounding effects age, BMI and M value were investigated using linear or semilog regression. Paired analyses (before vs after surgery) were performed with paired t-test. Analyses were done using JMP version 13.0 (SAS Institute, Cary, NC, USA). A *p* value ≤0.05 was considered statistically significant. Figures were created using ggplot package on *R* Studio.^38^

### Study approval

The protocol was approved by the Ethics Committee of the Hospital District of Southwestern Finland (ETMK 52/2018, NCT04343469), and the studies were performed according to the principles of the Declaration of Helsinki of 1975 (and as revised in 1983). All subjects gave written informed consent before any study procedures.

## Results

### Fasting brain ^1^H-MRS: effect of obesity and weight loss on brain metabolites (dataset A)

A total of 27 subjects with severe obesity (OBE) and 21 age- and sex-matched healthy, lean controls (CON) were evaluated in the fasting state. The two groups were well matched by age and sex. OBE subjects had impaired insulin sensitivity and glycemic control, along with higher fasting insulin and triglyceride levels, CRP and blood pressure (Table 2).

**Table 2.** Anthropometric and biochemical characteristics and brain metabolites of the study participants in dataset A.

	OBE (N = 27)	CON (N = 21)	*p* value
Men/women	7/20	9/12	0.22
Age (years)	51 [16]	44 [15]	0.16
NGT/IGT±IFG/T2D	10/10/7	20/1/0	<0.001
BMI (kg/m^2^)	40.7 ± 5.1	24.6 ± 2.1	<0.001
W/H	0.95 [0.18]	0.84 [0.15]	0.006
HbA_1c_ (mmol/mol)	38 [4]	34 [4]	0.003
Fasting plasma glucose (mmol/L)	5.7 ± 0.8	5.1 ± 0.4	0.002
Fasting plasma insulin (pmoL/L)	108 [42]	42 [21]	<0.001
Matsuda-ISI	6.9 ± 2.8	2.9 ± 1.6	<0.001
Systolic BP (mmHg)	138 ± 16	120 ± 11	<0.001
Diastolic BP (mmHg)	85 ± 9	76 ± 8	<0.001
Total cholesterol (mmol/L)	4.2 ± 0.7	4.3 ± 0.8	0.69
HDL cholesterol (mmol/L)	1.2 ± 0.2	1.5 ± 0.4	0.004
LDL cholesterol (mmol/L)	2.7 ± 0.7	2.6 ± 0.8	0.64
Triglycerides (mmol/L)	1.4 ± 0.5	1.0 ± 0.5	0.005
C-reactive protein (mg/L)	3.1 [3.0]	0.7 [0.7]	<0.001
Brain Glx (mM)	9.35 ± 1.35	9.60 ± 1.29	0.47
Brain myo-inositol (mM)	4.64 ± 0.53	4.57 ± 0.37	0.70
Brain total NAA (mM)	10.22 ± 0.72	10.57 ± 0.49	0.03
Brain total creatine (mM)	7.24 ± 0.40	7.21 ± 0.41	0.58
Brain total choline (mM)	1.11 ± 0.12	1.09 ± 0.12	0.36

Entries are mean ± SD or median [interquartile range]. NGT: normal glucose tolerance; IGT ± IFG: impaired glucose tolerance with or without impaired fasting glucose; T2D: type 2 diabetes; W/H: waist-hip ratio, Glx: glutamine/glutamate; NAA: N-acetyl-aspartate.

Of the brain metabolites assessed with 1H-MRS, only total NAA concentrations were statistically different between the two groups, with the subjects in the OBE group having lower total NAA concentrations (p = 0.05) (Table 2). When analyzing the data of all subjects (N = 48), higher age (β=-0.32, p = 0.029) rather than BMI (p = 0.16) or peripheral insulin sensitivity (Matsuda-ISI, p = 0.55) was associated with lower total NAA.

**Figure 3.**
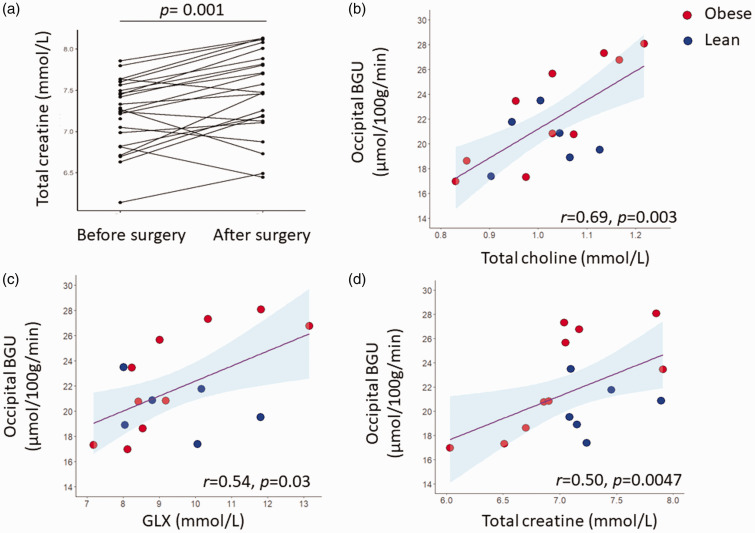
Total creatine levels were increased following weight loss (a). Under hyperinsulinemic, euglycemic clamp conditions, brain glucose uptake (GU) correlated positively glutamine/glutamate (Glx) (b), total choline (c), and total creatine (d) levels. The occipital region of interest was extracted from the brain glucose uptake data.

23 subjects with obesity were re-studied one year following bariatric surgery. They had achieved significant weight loss, losing on average 9 units of BMI, and significantly improved their insulin sensitivity as shown by the increase in the Matsuda-ISI (2.99 ± 1.54 before surgery *vs* 5.96 ± 2.55 after surgery, *p = *0.0006). All measured brain metabolites (total creatine, total choline, NAA, glutamine/glutamate [Glx]) showed a numerical increase following weight loss, but only total creatine concentration was significantly higher postoperatively (change 0.25 ± 0.32 mM, *p = *0.001) (Figure 3(a) and Supplemental Table 1).

### Paired brain ^1^H-MRS: effect of insulin on brain metabolites (dataset B)

The anthropometric and biochemical characteristics of the 11 OBE and 10 CON from dataset A to undergo the 1H-MRS studies also during hyperinsulinemic, euglycemic clamp (dataset B) were similar to the whole dataset A (data not shown). During the clamp studies, plasma insulin concentrations were raised quickly and were kept steady at 594 ± 103 pmol/L with no significant difference between OBE and CON groups. Especially in the OBE group plasma glucose was higher during fasting than under controlled euglycemia (Table 3), but plasma glucose levels or change in plasma glucose did not correlate with concentrations of brain metabolites or changes in their concentrations (p values above 0.34).

**Table 3.** Comparison of the fasting and insulin clamp metabolite concentrations per group, and intervention (N = 21) (dataset B).

	OBE (N = 11)		CON (N = 10)		*p*_group_	*p*_condition_	*p*_g*c_
	Fasting	Clamp	Fasting	Clamp			
Glx (mM)	9.86 ± 1.51	9.63 ± 0.66	9.56 ± 1.63	9.75 ± 1.06	0.83	0.54	0.94
Myo-inositol (mM)	4.81 ± 0.53	5.01 ± 0.45	4.52 ± 0.32	5.22 ± 1.24	0.88	0.04*	0.25
Total NAA (mM)	10.05 ± 0.61	9.79 ± 0.54	10.56 ± 0.42	10.58 ± 0.37	0.0007*	0.38	0.32
Total creatine (mM)	7.30 ± 0.30	7.19 ± 0.35	7.19 ± 0.44	7.34 ± 0.42	0.90	0.77	0.11
Total choline (mM)	1.15 ± 0.09	1.14 ± 0.09	1.08 ± 0.14	1.11 ± 0.16	0.36	0.59	0.26
PG (mmol/L)	5.7 ± 0.8	5.1 ± 0.3	5.2 ± 0.5	5.1 ± 0.2	0.11	0.036*	0.22
P Insulin (pmol/L)	90 [48–138]	540 [492–630]	42 [23–54]	498 [426–534]	0.05	<0.0001*	0.81
S FFA (mmol/L)	0.61 [0.47–0.86]	0.08 [0.04–0.22]	0.60 [0.32–0.67]	0.05 [0.03–0.06]	0.14	<0.0001*	0.58

Entries are mean ± SD or median [interquartile range]. Glx: glutamine/glutamate; NAA: N-acetyl-aspartate; PG: plasma glucose; P Insulin: plasma insulin; S FFA: serum free-fatty acids. PG, P Insulin and S FFA values during clamp are mean values of the measurements performed during the clamp.

In the paired fasting and clamp comparison, myo-inositol was the only metabolite which was significantly affected by insulin, with a similar increase in both lean and obese individuals (Table 3). Total NAA, total choline and total creatine showed no significant changes in response to insulin (Table 3).

### Associations between ^18^F-FDG-derived glucose uptake and ^1^H-MRS metabolites during hyperinsulinemia (dataset C)

The subject characteristics of Dataset C were similar to Dataset A and they are summarized in Supplemental Table 2, but only female subjects were included to diminish the possible confounding effect of sex in a small sample. In accordance with our previous studies, lower M-value/FFM (β = −0.74, p = 0.001) and younger age (β = −0.51, p = 0.042) predicted increased brain GU, whereas BMI had no significant effect (β = −0.41, p = 0.12).

During insulin-stimulation, brain GU correlated positively with Glx (r = 0.54, p = 0.032), total choline (r = 0.69, p = 0.003) and total creatine (r = 0.50, p = 0.047) concentrations (Figure 3(b) to (d)). These associations were driven by the OBE group (in the OBE: r = 0.79, p = 0.006, r = 0.83, p = 0.003, and r = 0.75, p = 0.01, for the association between BGU with GLX, total choline, total creatine respectively, whereas in the CON: r = −0.33, p = 0.5; r = 0.05, p = 0.9; r = 0.14, p = 0.8, for the association between BGU with GLX, total choline, total creatine respectively). Myo-inositol (r = 0.46, p = 0.07) and NAA (r = 0.35, p = 0.18) concentrations showed no statistically significant association with BGU.

## Discussion

The strength of the current study is based on the novel approach combining two complementary methods, ^18^FDG-PET and ^1^H-MRS, to characterize the effects of obesity on brain glucose metabolism in humans *in vivo*. While it is not possible to establish causal relationships with the applied methods, the results highlight the role of insulin and glucose metabolism in alterations in brain metabolites often seen in obesity.

First, in the fasting state, we assessed whether there are any baseline differences between neurologically healthy subjects with obesity and their age and sex-matched lean controls. We found only total occipital NAA concentrations to be lower in subjects with obesity, which reproduces the findings from parietal, prefrontal and fontal cortex as well as hippocampus from previous studies in insulin resistant or obese subjects.^37–40^ As NAA is synthesized almost exclusively in neurons in the brain,^41^ and decreased concentrations have been associated with aging and several neurodegenerative diseases,^42–46^ lower NAA concentrations have been attributed to neuronal damage in obesity. Additionally, neuronal damage has been suggested to manifest the concomitant reduction of white and gray matter volumes.^47^

Apart from the difference in total NAA concentrations in the fasting state, the two groups had similar concentrations of the other brain metabolites assessed. This is in contrast to a study by Gazdinski *et al.*, where the investigators reported also lower choline concentrations in frontal white matter (WM) with increasing BMI^18^. As the authors argued, the frontal WM myelinates later than the other lobes, thus the apparent discrepancy between our results and those of that study could be attributed to different characteristics of the examined lobes. A meta-analysis from Wu *et al*. reported that the myo-inositol/creatine levels were increased in the occipital lobe in subjects with T2D compared to healthy lean controls.^44^ However, as the authors acknowledged their results should be interpreted carefully since there was heterogeneity in the studies included. In the currents study, we would attribute the lack of increased myo-inositol levels in the OBE group either to their good glycemic control, or to differences between the studied brain regions, or both.

We also performed paired brain ^1^H-MRS experiments to assess the acute effect of insulin on brain metabolites. While one previous study^24^ found that insulin infusion resulted in an increase in frontal NAA/creatine and frontal and temporal Glx/creatine and a decrease in frontal choline/creatine and temporal choline/H_2_O and myo-inositol/H_2_O, in our data 2-hours of euglycemic hyperinsulinemia changed only brain myo-inositol concentrations, which were increased during the clamp similarly in lean and obese subjects (Table 3).

Myo-inositol is more concentrated in glial cells,^23^ where it serves as a vital precursor to several intracellular second messengers downstream from the insulin receptor in the phosphoinositide 3-kinase/protein kinase B (AKT)-signaling arm.^48^ As the brain has the capacity to convert D-glucose into myo-inositol,^49^ the increase in the latter might occur in response to accumulation of intracellular glucose-6-phosphate. In contrast, brain myo-inositol concentration in the anterior cingulate cortex, but not the occipital cortex, has been shown to decrease when insulin is administered to younger healthy, lean subjects.^24,29^ The discrepancy with the current results could be explained with a different selection of region, as well as impaired insulin sensitivity and older age, as brain myo-inositol levels have been shown to increase with age and also early on in the development of cognitive impairment.^50^ Therefore the current study does not yield definitive support for or against cortical astrocytosis in obesity; for one, myo-inositol is an indirect marker of supposed gliosis, and might not correlate with histological findings or neuroinflammation,^51,52^ and second, results from our study emphasize the importance of the cerebral metabolic milieu in interpreting ^1^H-MRS metabolite results.

A salient finding of the present study is that brain glucose uptake under insulin clamp was positively related to glutamine/glutamate (Glx), total creatine, and total choline levels in the occipital lobe. Glutamate is the major excitatory neurotransmitter; once released in the synapses it is quickly cleared by the astrocytes, and transformed in glutamine which is then cycled back to neurons.^53^ Previous studies have shown that Glx increases in response to insulin stimulation^43^ and to hyperglycemia in subjects with type 1 diabetes, independently of neuronal activity to induce enhanced glutamate-glutamine cycling.^25^ The increase also occurs postprandially in rats and healthy human subjects, with the increase in glutamate synthesis possibly being mediated by the increase in plasma glucose.^54^ While we observed no effect of severe obesity on fasting Glx levels or changes during hyperinsulinemic euglycemia, brain Glx correlated with insulin-stimulated brain GU. While increased intracellular glucose concentration in astrocytes might stimulate glutamate synthesis, enhanced glycolysis is also required to provide glutathione to maintain the redox status in association with upregulated glutathione/glutamine cycling.^51^ Additionally, impaired further metabolism of glutamate and glutathione could result in their accumulation, as reported in *db/db* mice.^53^

Choline, representing different choline-containing compounds, is considered a marker of membrane turnover. As the concentration of choline is higher in glial cells than neurons,^23^ and ^1^H-MRS studies have demonstrated increased concentrations in different inflammatory states,^55^ it is often considered also a sign of gliosis. In the current data, brain choline did not change in response to insulin but was associated with BGU. It could therefore be argued that increased glucose uptake reflects increased number of astrocytes or altered membrane phospholipid metabolism.

Finally, total brain creatine was also positively associated with BGU. Brain creatine levels have previously been shown to be increased in metabolic syndrome during fasting,^56^ and in subjects with type 1 diabetes during hyperglycemia.^25^ Because the ^1^H-MRS signal for total creatine represents both creatine and phosphocreatine, it is not possible to detect whether energy storing as phosphocreatine is increased in response to insulin-stimulation; however, the higher concentration seen in insulin resistance may imply an increased need for a readily available energy buffer for large fluctuations in energy needs, as seen in astrocytes.^57^ Once again, even though creatine and phosphocreatine (*i.e.,* total creatine) are found in all neural cells, their astrocytic concentration is twice that of neurons.^58^

In our previous studies focusing on brain metabolism, we have shown that insulin-stimulated BGU is normalized already 6 months after bariatric surgery,^14^ and therefore we expected to see changes in ^1^H-MRS metabolites possibly associated with glucose metabolism as well. Recently Bottari et al., reported a decrease in choline-containing compounds and myo-inositol one year after bariatric surgery, whereas levels of NAA, creatine or Glx remained unaffected.^59^ This in contrast to our results only showing an increase in total creatine. However, as Bottari et al. did not report the condition the scans were performed in, it is difficult to make a comparison between the studies. An earlier study has shown that brain water content is increased in obesity,^60^ and therefore it is plausible that the increase in brain metabolites following weight loss could be driven by a decrease in brain water content, which we were unfortunately unable to quantitate from the performed scans.

Taken together, this is the first study to link directly upregulated brain glucose uptake during euglycemic hyperinsulinemia with increased concentrations of glutamine/glutamate, total choline, and total creatine in humans *in vivo*. This supports the growing evidence in both animal models and humans of altered TCA cycling in the brain associated with obesity and insulin resistance,^54,61,62^ with the lower brain glucose concentrations of a previous ^1^H-MRS study possibly explained by a higher turnover of glucose into further metabolites.^28^ While several of these findings might be attributed to astrocytosis, they do not confirm the existence of central inflammation *per se*. Rather, our results emphasize how understanding metabolic disruption is essential to further characterize these phenomena.

While the established and sensitive methodology used in the current study is a strength, it also poses some limitations due to the radiation burden and cost of the studies, which limit the possibility of repeat studies in the same subjects. The small sample sizes, and recruiting only female participants in dataset C, also limit the ability to generalize the results. Another important consideration is that because of the conventional ^1^H-MRS protocol applied, brain glucose concentrations could not be assessed. In this setting, the signal of brain glucose consists of multiple peaks that overlap with other metabolites. To overcome this problem, a recent elegant study assessing the effect of obesity and T2D on brain glucose concentrations applied hyperglycemic clamps to increase the brain glucose signal, and then subtracted the baseline brain glucose peaks from those of the hyperglycemic state.^28^ Finally, from a technical point of view the water suppression method differed in the two scanner models. CHESS with a small bandwidth was used on Siemens while excitation method with a bandwidth of 200 Hz was used on Philips. On Siemens this could have led to a larger water residual while the broad bandwidth on Philips might have affected the amplitude of some of the metabolite signals close to water. Due to this reason, absolute values of metabolites concentrations from different scanners and datasets are not directly comparable.

To conclude, using a novel approach applying two complementary imaging modalities, we were able to show for the first time that during insulin-stimulation, cerebral glucose uptake is coupled with concentrations of glutamine/glutamate, choline, and creatine. Of the studied metabolites, myo-inositol was the only metabolite to show a consistent increase in concentration from fasting to hyperinsulinemia, and in a similar degree in lean and obese subjects. We hypothesize that the coordinated changes are driven by upregulated brain metabolism in response to insulin stimulation, but more studies in vivo in humans would be warranted to consolidate the present findings and to elucidate the mechanisms involved in central insulin resistance.
